# The Concept of Venous Steal: The Impact of Vascular Stenosis and Outflow Pressure Gradient on Blood Flow Diversion

**DOI:** 10.3390/medicina61040672

**Published:** 2025-04-06

**Authors:** Mindaugas Pranevičius, Dalius Makackas, Andrius Macas, Kęstutis Petrikonis, Gintarė Šakalytė, Osvaldas Pranevičius, Rimantas Benetis

**Affiliations:** 1 Lithuanian University of Health Sciences, 50162 Kaunas, Lithuania; osp9003@med.cornell.edu; 2 Department of Applied Informatics, Faculty of Informatics, Kaunas University of Technology, 50254 Kaunas, Lithuania; dalius.makackas@ktu.lt; 3 Department of Anesthesiology, Lithuanian University of Health Sciences, 50162 Kaunas, Lithuania; andrius.macas@lsmu.lt; 4 Department of Neurology, Lithuanian University of Health Sciences, 50162 Kaunas, Lithuania; kestutis.petrikonis@lsmu.lt; 5 Institute of Cardiology, Lithuanian University of Health Sciences, 50162 Kaunas, Lithuania; gintare.sakalyte@lsmu.lt; 6 Department of Heart, Lithuanian University of Health Sciences, 50162 Kaunas, Lithuania; rimantas.benetis@lsmu.lt

**Keywords:** arterial stenosis, venous drainage, Starling resistor model, compartment pressure, segmental perfusion pressure, venous steal, arterial steal, subendocardial ischemia, selective cerebral perfusion, regional blood flow

## Abstract

Vascular steal refers to the diversion of blood flow between collateral vessels that share a common inflow restricted by arterial stenosis. Blood is diverted from the high-pressure to the low-pressure, low-resistance system. Vascular steal is associated with anatomical bypass or vasodilation in the collateral network and is called “the arterial steal”. However, we have demonstrated that in the presence of an outflow gradient (e.g., intra-extracranial), blood is shunted to a lower pressure system, a phenomenon we term “venous steal”. Using Thevenin’s equivalent, we generalized the concept of venous steal to apply it to any region of the vascular system with increased outflow pressure. Both arterial steal, caused by increased collateral network conductivity, and venous steal, resulting from lower collateral outflow pressure, reduce compartment perfusion. This occurs indirectly by increasing flow and the pressure gradient across the arterial stenosis, lowering the segmental compartment perfusion pressure—the difference between post-stenotic (inflow) and compartmental (outflow) pressures. Venous steal diverts blood flow from compartments with elevated pressure, such as intracranial, subendocardial, the ischemic core, and regions of focal edema due to inflammation, trauma, or external compression. In shock and low-flow states, it contributes to regional blood flow maldistribution. Treatment of venous steal addresses inflow stenosis, increased compartmental pressure and systemic loading conditions (arterial and venous pressure) to reverse venous steal malperfusion in the ischemic regions.

## 1. Introduction

While arterial determinants of regional blood flow are well known, factors affecting venous outflow are much less studied and are commonly ignored. We generalized the Starling resistor model to describe regional blood flow redistribution due to the outflow pressure differences to make it applicable to any regional vascular network.

Regional blood flow distribution depends on:

- Systemic pressure (Pa),
- Arterial stenosis,
- Regional and collateral network conductivities (G, G__COLLATERAL_),
- Venous outflow pressure (Pv).

In most tissues, except the most superficial ones, extravascular pressure (P_ext) exceeds systemic venous pressure (Pv). Thus, local tissue pressure (P_ext), rather than systemic venous pressure, governs regional venous outflow [1,2,3,4].

Arterial stenosis creates a regional inflow pressure gradient (Pa − Pd), so post-stenotic pressure (Pd), not arterial pressure (Pa), determines inflow pressure. In compartments with increased tissue pressure (e.g., intracranial or subendocardial), regional tissue perfusion is determined by the segmental perfusion pressure (SPP = Pd − P_ext). SPP is lower than perfusion pressure (PP = Pa − P_ext) due to the pressure drop (Pa − Pd) [5,6].

The classical definition of compartment perfusion pressure (Pa−P_ext), such as cerebral perfusion pressure, does not account for reduced perfusion due to redistribution, which increases the Pa−Pd gradient.

To address arterial stenosis, increased compartment pressure, collateral network size, and the outflow pressure gradient between the compartment and collateral network, SPP (Pd−P_ext) must be used as the driving gradient for compartmental perfusion rather than systemic gradient (Pa−P_ext).

Although systemic venous pressure (Pv) does not directly affect compartment outflow when Pv < P_ext, it determines collateral outflow and, consequently, the pressure drop (Pa−Pd) across the inflow stenosis. Lower Pv in the collateral system reduces post-stenotic compartment perfusion due to collateral outflow diversion, driven by increased outflow pressure gradient (P_ext−Pv), when Pv falls below P_ext. We termed this phenomenon “the venous steal”—a venous pressure-dependent collateral blood flow diversion [5,6].

## 2. Compartmental Perfusion Model

Using the waterfall analogy, compartment perfusion is described by compartment perfusion pressure (PP = Pa−P_ext). Perfusion is likened to water flowing over waterfall: when venous pressure (Pv) is lower than external pressure (P_ext), Pv does not influence the flow through the compartment, just as the height drop beyond a waterfall does not affect the flow over it. For example, cerebral perfusion pressure is defined as the difference between arterial and intracranial pressure (Pa-ICP). Similarly, myocardial perfusion pressure is the difference between coronary and myocardial pressure during diastole (Pa-P_myocardial). In cases of arterial stenosis, segmental perfusion pressure (SPP = Pd − P_ext) where Pd represents the distal pressure after stenosis, provides a more appropriate determinant of perfusion. However, collateral flow diversion must be considered, as it impacts compartment flow, similar to how a river branch diverts water and reduces flow over a waterfall. Just as a waterfall’s flow halts when the water level drops below its crest, a decrease in downstream pressure Pv can divert flow to collateral pathways, potentially halting compartment flow.

Perfusion in parallel compartments has been modeled using a 0-D Starling resistor model [5]. The influence of collateral outflow on segmental compartment perfusion pressure has been examined using a Thévenin equivalent of circulation in an intra-extracranial flow distribution model [6].

**Figure 1:**
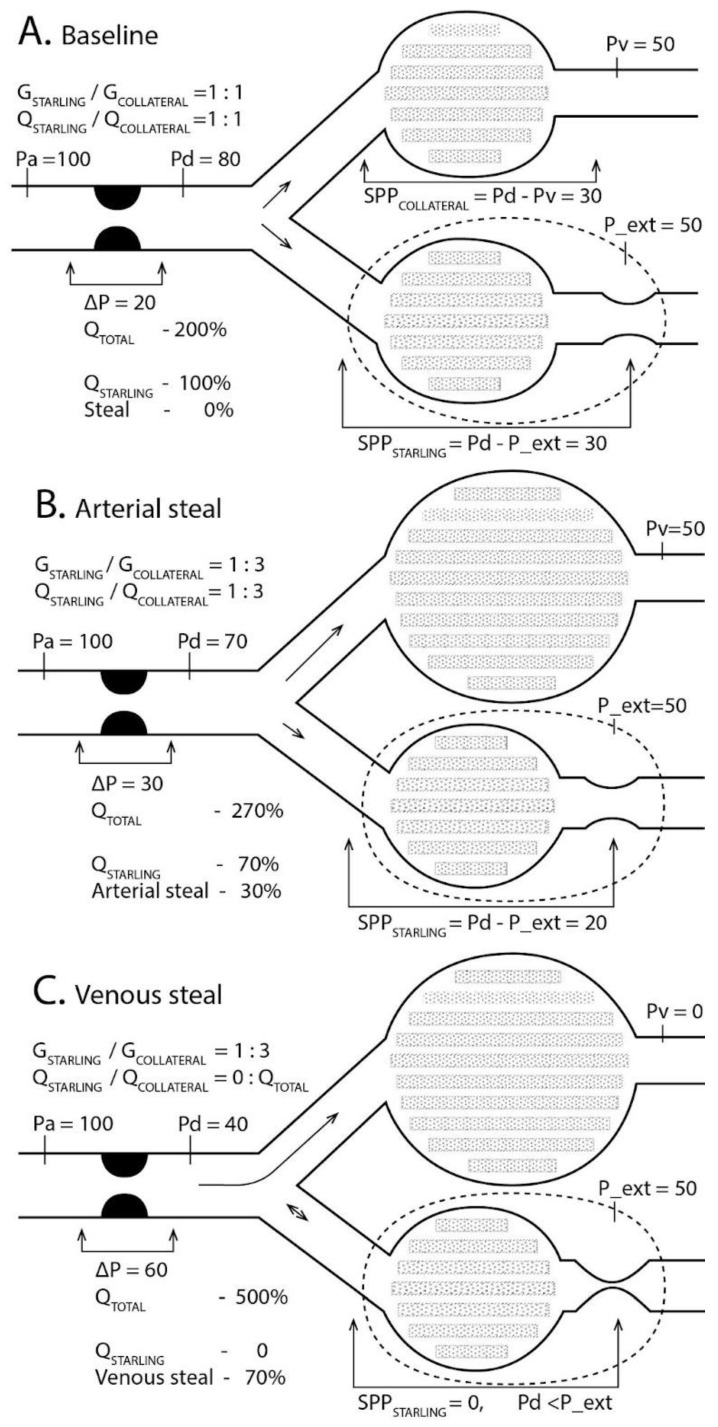
Regional blood flow distribution between Starling compartment and collateral outflow. (**A**)**. Baseline**: Flow is evenly distributed between the Starling resistor and collateral outflow when their conductances and outflow pressures are equal. (**B**)**. Arterial Steal**: Collateral vasodilation increases collateral conductance G__COLLATERAL_. Segmental perfusion pressure decreases due to the increased pressure drop ΔP across the stenosis. Outflow pressures Pv and P_ext are equal, so the segmental perfusion pressures through both the compartment (SPP__STARLING_ = Pd−P_ext) and collaterals (SPP__COLLATERAL_ = Pd−Pv) remain equal. (**C**)**. Venous Steal**: Reduced venous pressure Pv increases collateral outflow and pressure drop ΔP across the stenosis. When Pd < P_ext, no flow occurs through the Starling resistor.

Regional blood flow can be represented by a 0-D electrical Starling resistor equivalent, featuring a common inflow and a variable conductance collateral outflow network (G__COLLATERAL_) (Figure 1). During ischemia, the vascular reserve of the Starling resistor is exhausted, fixing its conductance (G__STARLING_) due to maximal vasodilation. Flow through the Starling resistor depends on the segmental perfusion pressure (SPP):

SPP = Pd − Pv, when Pv > P_ext,

SPP = Pd−P_ext, when P_ext ≥ Pv.

The flow distribution effect on inflow pressure Pd is calculated using Ohm’s and Kirchhoff’s laws [5,6,7]:

Pd = (Pa×G_inflow + Pv×G__COLLATERAL_ + P_ext×G__STARLING_) / (G_inflow + G__COLLATERAL_ + G__STARLING_).

To illustrate pressure distribution in relative terms, we set Pa = 100, P_ext = 50, and varied Pv from 0 to 100 (relative to Pa). Conductance of the Starling pathway (G__STARLING_) was assumed to be 1, while collateral conductance G__COLLATERAL_ ranged from 0 to 5.

The vascular compartment with increased pressure is modeled as a Starling resistor. At baseline, flow is evenly distributed between Starling resistor and collateral outflow (A). Arterial steal diverts blood flow to the collateral pathway due to the vasodilation (B). Decreasing venous pressure enhances collateral outflow, stopping flow in the Starling resistor due to the venous steal (C). Adapted from [5,8]. Interactive model is available at: https://sites.google.com/view/venous-steal (Supplementary Materials, URL accessed on 29 March 2025).

Variables: Pa, aortic pressure; Pd, arterial (inflow) pressure distal to the stenosis or its Thévenin equivalent; P_ext, extravascular compartment pressure; G__STARLING_, compartment vascular conductance (fixed at maximal 1 due to vasodilation); G__COLLAT_, collateral outflow conductance, increasing during arterial steal due to the vasodilation (B); Pv, venous pressure; Q__STARLING_, blood flow through the Starling resistor; proportional to SPP__STARLING_ = Pd−P_ext when P_ext > Pv, as P_ext determines effective outflow pressure due to venous compression. Pressures and flows are normalized (Pa = 100, Q__STARLING_ = 100). Arterial steal reduces, while venous steal ceases flow, resulting in a “no flow/no reflow” state (Q__STARLING_ = 100, 70, 0 in Figure 1A–C).

**Figure 2:**
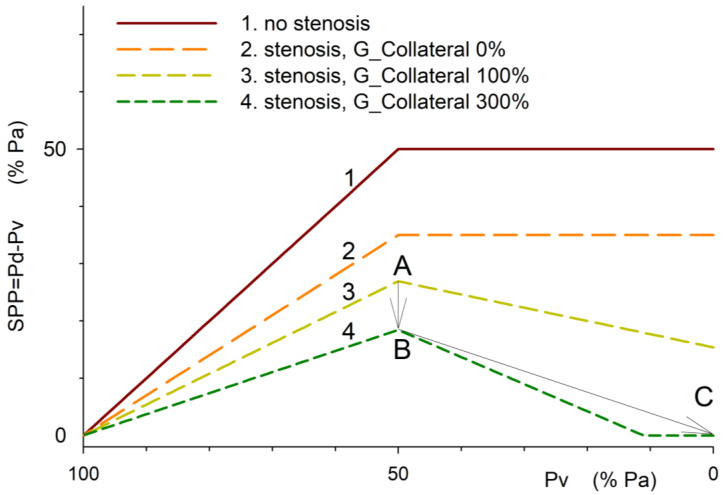
Dependence of segmental perfusion pressure (SPP) on venous pressure (Pv) in a compartment with increased pressure P_ext = 50: without arterial stenosis (1), with arterial stenosis (2), and with increasing collateral outflow conductance (3, 4). Venous pressure on *X*-axis decreases from arterial (100%) to zero left to right. Points A-C correspond to Figure 1A-C. Interactive simulation is at https://sites.google.com/view/venous-steal. (Supplementary Material, URL accessed on 29 March 2025).

SPP, the difference between Pd and Pv, increases as Pv decreases, while Pv exceeds P_ext (100 > Pv > 50). With arterial stenosis and collateral outflow (Figure 2, graphs 3, 4) increasing, G__COLLATERAL_ raises flow through the stenosis, reducing Pd and SPP due to arterial steal (2→3→4, 100 > Pv > 50). When Pv < P_ext, reduced Pv enhances collateral, but not compartmental outflow, lowering Pd, SPP, and perfusion due to venous steal (Figure 2, graphs 3, 4, 50 > Pv > 0).

Points 3A, 4B, and 4C correspond to SPP in Figure 1A–C. Both arterial and venous steal reduce SPP by increasing collateral outflow across the stenosis. Venous steal, unlike arterial steal, can completely stop flow in a compartment with elevated P_ext when Pd < P_ext (“no flow”, 4C).

## 3. Starling Resistor

Starling used a collapsible tube in a compartment with adjustable pressure (P_ext) to simulate systemic circulation while studying cardiac contractility in an isolated heart preparation [9]. This setup, later called the Starling resistor, shows that blood flow (Q__STARLING_) is proportional to the segmental perfusion pressure- (SPP__STARLING_ = Pd-P_ext) and is independent of venous pressure when P_ext > Pv.

Apart from the whole systemic circulation, the Starling resistor is used to describe brain, lung, and myocardial perfusion, their perfusion pressures, as well as boundary conditions in 3D vascular models [2,4,10,11].

## 4. Blood Flow Distribution in Parallel Starling Resistors with Common Inflow

The Starling resistor can model the entire circulation and individual segments. We used it to study blood flow redistribution between two parallel compartments [5,6,7]. This model conceptualizes blood flow redistribution between a compartment with elevated extravascular pressure, and the rest of systemic circulation. Using Thévenin equivalent generalizes the findings of flow distribution in compartments with an anatomically distinct common inflow (such as in common carotid artery or left main coronary artery) to any micro- or macro-circulatory segment with increased P_ext, as any point of the circulation can be mapped to an arterial inflow with distinct compartmental and collateral outflows.

Generalized venous steal concept is applicable to any collateral network with heterogeneous outflow pressures, including subendocardial and subepicardial layers in the heart.

## 5. Arterial and Venous Steal

Vascular stenosis and increased extravascular pressure create conditions for arterial and venous steal, increasing collateral outflow.

Venous steal diverts blood from areas with elevated tissue pressure to the collateral network. Decreasing venous pressure (Pv) increases collateral outflow, and inflow pressure gradient (Pa−Pd). In a compartment with increased tissue pressure (P_ext), outflow pressure, set by P_ext, remains constant despite Pv reduction, while inflow pressure (Pd) decreases.

When the vascular flow reserve is exhausted by maximal vasodilation, resistance is fixed and blood flow is proportional to SPP. Without stenosis, SPP equals the systemic perfusion pressure (PP = Pa −- Pv). With stenosis, SPP decreases to Pd −- Pv (Figure 1C and Figure 2: 1→2). Increased collateral outflow through a shared stenosis reduces Pd (arterial steal—Figure 2, 2→3→4, 100 > Pv > 50). When Pv < P_ext, outflow pressure is determined by P_ext (SPP = Pd −- P_ext). If Pv in the collateral segment falls below P_ext, collateral flow increases, further reducing SPP due to the increased flow and pressure drop (Pa−-Pd) (Figure 2, graphs 3–4, 50> Pv >0).

Per Ohm’s and Kirchhoff’s laws, blood flow through the parallel segments is proportional to their SPP, and shared inflow pressure (Pd) depends on the total blood flow. Increased collateral outflow increases the pressure drop through the common inflow. In arterial steal, collateral flow rises due to vasodilation, in venous steal, it increases due to selective venous outflow enhancement when Pv < P_ext.

## 6. Venous Steal Conditions During Shock and Anesthesia

Circulatory shock may include regional flow maldistribution due to venous steal with outflow pressure gradients, especially in hypovolemia. Similarly, most anesthetics induce vasodilation, reducing the autoregulatory reserve, which may cause venous steal malperfusion in susceptible regions (e.g., subendocardial ischemia with coronary stenosis).

Low arterial pressure—due to drugs, anesthesia, or shock—predisposes to venous steal, as outflow pressure heterogeneity becomes more significant relative to arterial pressure (Pa assumed 100% in this simulation).

Autoregulation compensates for venous steal. In low-flow states (e.g., shock or deep anesthesia), the vascular network may be maximally vasodilated. Thus, any collateral blood flow diversion due to arterial or venous steal reduces the compartmental perfusion pressure and flow. Venous steal may lead to regional no-flow states, like “mottled skin” in shock, or no-reflow post-revascularization, potentially reversible with volume or inflow pressure correction.

## 7. The Significance of Venous Steal and Reverse Venous Steal as a Therapeutic Strategy

Venous steal may contribute to malperfusion in compartments with increased pressure. Venous steal can be reversed by correcting SPP components (inflow and outflow pressures). Perfusion in regions with increased tissue pressure is determined by the local inflow and outflow pressures—these regions “do not know” the systemic pressure in the aorta. Systemic arterial pressure, while being very important, is not the sole factor of regional perfusion: regional perfusion depends on inflow stenosis, outflow pressure, and interaction between collateral networks.

The venous steal concept and its contribution to the segmental regional perfusion pressure establishes a rational framework for regional blood flow optimization and introduces the possibility of new therapeutic modalities, like controlling of intra-extracranial blood flow distribution during selective brain cooling on cardiopulmonary bypass.

### 7.1. Quantification of Arterial and Venous Steal

The ischemic compartment and collateral flow define the pressure drop (ΔP = Pa − Pd) across the common inflow. Occluding collateral circulation allows the assessment of isolated compartment perfusion (Figure 2, graphs 1, 2) and the arterial and venous steal components (Figure 2, graphs 3, 4). Maximally dilating the collateral circulation and reducing Pv below P_ext evaluates venous steal’s impact. When these boundary conditions are unachievable, the influence of venous steal is assessed dynamically by altering G_collateral, Pv, or P_ext in order to estimate Pd and SPP [12]. This calculates the venous steal fraction as a reduction in regional perfusion (Figure 2: SPP drop from 18 to 0, B→C indicates 100% venous steal).

### 7.2. Post-Stenotic Pressure Pd and Its Relationship with Arterial Pressure (FFR)

Small vessels “do not know” the systemic pressure in the aorta. Thus, in compartments with arterial stenosis and increased tissue pressure, perfusion depends on segmental, not systemic perfusion pressure. In cerebral circulation, Pd (distal to extracranial stenosis) is measured via catheterization or as carotid stump pressure during carotid endarterectomy. Non-invasive methods for measuring Pd include plethysmography or directional Doppler ultrasound [13,14,15,16,17]. In coronary circulation, Pd is measured as the fractional flow reserve (FFR) [8,18]. Due to transmural venous steal, Pd depends not only on stenosis severity but also on intramyocardial pressure (P_ext) and coronary sinus pressure (Pv). When Pv < P_ext, the gradient (Pa−Pd) increases due to venous steal. This affects FFR values, requiring considering the transmural pressure gradient between the endocardium and epicardium and when interpreting FFR.

### 7.3. Estimating Compartment Pressure P_ext

In the brain, venous outflow pressure is determined by intracranial pressure (ICP), which can be assessed non-invasively via ultrasound [19,20] or ophthalmoscopy by observing venous collapse in the ocular fundus [21]. Another method measures blood flow redistribution between extra- and intracranial compartments via the ophthalmic artery using an inflatable supraocular cuff. This approach, pioneered by researchers from Lithuania, demonstrated that equalizing cuff pressure with ICP balances the pulsatile blood flow in the ophthalmic artery, the collateral pathway between intracranial arteries and supraorbital artery, and can be used to estimate ICP [6,22].

### 7.4. Segmental Perfusion Pressure (SPP) for Intracranial Compartment

Compartment perfusion depends on the SPP (Pd −- P_ext). Due to the extracranial stenosis and intra-extracranial blood flow redistribution with increased ICP, segmental intracranial perfusion pressure differs from cerebral perfusion pressure [6]:

SPP_intracranial = Pd − ICP = FFR × CPP − G_extracranial (1 − FFR)(ICP − P_ext),

where Pa is arterial pressure, ICP is intracranial pressure, Pd is the mean pressure in the Circle of Willis, G_extracranial is relative extracranial conductance, and P_ext is extracranial outflow pressure. This formula accounts for the extracranial stenosis (FFR = Pd/Pa) and intra-extracranial blood flow diversion due to the venous steal (second term).

### 7.5. Reverse Venous Steal as a Therapeutic Strategy

In low-flow conditions (e.g., penumbra surrounding core with increased tissue pressure), reverse steal may occur if P_ext < collateral drainage pressure (P_ext_Collateral). This has been observed between extra- intracranial compartments, where flow through the supraorbital artery reverses direction during carotid cross-clamping and may be augmented by extracranial outflow manipulation [6,23]. Likewise, retrograde cardioplegia has been shown to decrease the need for inotropic support when administered together with antegrade cardioplegia [24]. This can enhance selective cerebral perfusion during cardiac bypass.

### 7.6. Enhancement of Selective Cerebral Cooling via Reverse Venous Steal

Selective cerebral cooling mitigates neurological consequences of global or regional cerebral ischemia [25,26]. Current intracranial cooling methods for a closed skull, however, do not generate significant temperature gradients between the brain and central circulation [27]. Adjusting the extra-intracranial drainage can direct cooled scalp blood intracranially, enhancing selective brain cooling [12].

### 7.7. Utilizing the Venous Steal Model to Optimize Revascularization Strategies

Revascularization strategies in myocardial ischemia should account for dynamic manifestations of coronary stenosis. The subendocardial venous steal model conceptualizes stenosis’s physiological impact on transmural myocardial flow distribution with different loading conditions and guide revascularization decisions. Patient-specific in silico models estimate FFR and the instantaneous wave-free ratio (iFR) before and after revascularization [28,29]. These models can be adapted to estimate transmural flow distribution.

### 7.8. Ensuring Adequate Cerebral Perfusion During Selective Antegrade Perfusion and Carotid Endarterectomy

During selective antegrade perfusion and carotid endarterectomy, blood flow redistribution through the Circle of Willis defines regional cerebral perfusion (rSPP). Combining computational modeling with preoperative imaging can determine the minimal predicted flow for specific regions under varying inflow and outflow pressures. These analyses can guide perfusion strategies, and optimize selective cerebral perfusion and cooling by reversing intra-extracranial gradients.

## 8. Discussion of Venous Steal Model Limitations

Blood flow distribution between ischemic and collateral regions depends on relative pressure and conductance. Regional blood flow distribution can be analyzed using the generalized Starling resistor analogy. While the Starling resistor concept is more than 100 years old, it provides a generalizable framework of blood flow distribution with external compression. Originally applied to cerebral circulation, the venous steal concept, as shown in this article, can be applied to any region of the circulation, lumping the rest of the vascular network into the inflow and collateral outflow Thevenin’s equivalents, presuming linear pressure/flow relationship. While such simplification allows conceptualization of regional blood flow distribution, detailed mapping of regional blood flow and its dependence on loading conditions requires a more complex approach utilizing the field of Starling resistors, with dynamic inflow and outflow interactions that may result in blood flow and volume oscillations. The applicability of regional blood flow redistribution to the clinical pathways of diagnosis and treatment of cardiovascular conditions remains to be established. With these limitations, the concept of venous steal can be used to assess the impact of vascular stenosis and outflow pressure gradient on blood flow diversion in every organ or compartment, including the heart, lungs, brain, abdominal viscera, and skeletomuscular compartments.

## 9. Conclusions

In compartments with elevated pressure, perfusion depends not only on systemic and compartmental pressures, but also on the inflow stenosis, conductivity of the collateral network, and the outflow pressure in the collateral network. The 0-D Starling resistor model with collateral outflow allows for conceptualizing regional blood flow optimization—compartment perfusion can be improved by increasing systemic pressure, dilating or revascularizing stenoses, and reducing compartment pressure or raising systemic venous pressure, depending on the clinical context and prevailing mechanism of regional ischemia.

## Data Availability

“Venous steal blood flow simulation” dataset is available at Mendeley Data, V1, [DOI: 10.17632/zkc963nr99.1] [https://data.mendeley.com/drafts/zkc963nr99] (accessed on 29 March 2025).

## Supplementary Materials

Segmental pressure equation can be downloaded at: https://static-content.springer.com/esm/art%3A10.1038%2Fs41598-021-85931-x/MediaObjects/41598_2021_85931_MOESM1_ESM.docx (accessed on 29 March 2025). Time-lapse animation, interactive model and chart of venous steal simulation is available at: https://sites.google.com/view/venous-steal (URL accessed on 29 March 2025).
